# Phylogeographic pattern of *Rhizophora* (Rhizophoraceae) reveals the importance of both vicariance and long-distance oceanic dispersal to modern mangrove distribution

**DOI:** 10.1186/1471-2148-14-83

**Published:** 2014-04-17

**Authors:** Eugenia YY Lo, Norman C Duke, Mei Sun

**Affiliations:** 1 Department of Ecology and Evolutionary Biology, University of California at Irvine, Irvine, CA 92697, USA; 2 Trop WATER, James Cook University, Townsville, QLD, Australia; 3 School of Biological Sciences, The University of Hong Kong, Pokfulam Road, Pokfulam, Hong Kong

**Keywords:** Atlantic-east pacific, Chloroplast DNA, Divergence time, Indo-west pacific, Nuclear markers, Long-distance dispersal, Mangroves, Phylogeography, Rhizophora, Vicariance

## Abstract

**Background:**

Mangroves are key components of coastal ecosystems in tropical and subtropical regions worldwide. However, the patterns and mechanisms of modern distribution of mangroves are still not well understood. Historical vicariance and dispersal are two hypothetic biogeographic processes in shaping the patterns of present-day species distributions. Here we investigate evolutionary biogeography of mangroves in the Indo-West Pacific (IWP) and western Atlantic-East Pacific (AEP) regions using a large sample of populations of *Rhizophora* (the most representative mangrove genus) and a combination of chloroplast and nuclear DNA sequences and genome-wide ISSR markers.

**Results:**

Our comparative analyses of biogeographic patterns amongst *Rhizophora* taxa worldwide support the hypothesis that ancient dispersals along the Tethys Seaway and subsequent vicariant events that divided the IWP and AEP lineages resulted in the major disjunctions. We dated the deep split between the Old and New World lineages to early Eocene based on fossil calibration and geological and tectonic changes. Our data also provide evidence for other vicariant processes within the Indo-West Pacific region in separating conspecific lineages of SE Asia and Australia-Pacific at the Oligocene-Miocene boundary. Close genetic affinities exist between extant Fijian and American lineages; East African and Australian lineages; and Australian and Pacific lineages; indicating relatively more recent oceanic long-distance dispersal events.

**Conclusions:**

Our study demonstrates that neither vicariance nor dispersal alone could explain the observed global occurrences of *Rhizophora*, but a combination of vicariant events and oceanic long-distance dispersals can account for historical diversification and present-day biogeographic patterns of mangroves.

## Background

Mangroves are key components of coastal ecosystems in tropical and subtropical regions worldwide [1,2]. More than 100 plant species are associated with mangrove vegetation, but only about 80 from 21 mostly angiosperm families are termed mangroves – being plants exclusive to mangrove habitats between mean sea level and the highest tide elevation. Their occupation of the tidal zone is manifested in a range of specialized attributes, including water-buoyant propagules that in some species can survive in seawater for long periods [3,4]. Environmental factors limit mangrove distributions are known to be primarily temperature and rainfall, and thus the most prolific mangrove occurrences are restricted to tropical and temperate latitudes in regions of high rainfall [2]. Albeit the subject of on-going research e.g., [5-8] the patterns and factors leading to modern distribution of mangroves are still not fully understood, particularly in the Indo-West Pacific Region. It is postulated that mangroves have dispersed widely, influenced by continental drift and other vicariant events, on top of species-specific long distance dispersal limited by land barriers and direction of ocean currents.

For example, despite comparable environmental conditions, mangrove species richness is dramatically higher in the Indo‒West Pacific (IWP; ~65 species of 23 genera) compared to the Atlantic East Pacific (AEP; ~15 species of 8 genera) [2]. A number of hypotheses have been proposed during the last century to explain these distributional differences [9-14], but the view of each author is best considered dated – with comparisons made under essential caveats. For instance, the hypothesis for long-distance dispersal and its ‘centre of origin’ concept [9,10] was proposed before the theory of continental drift was generally accepted, when molecular genetics was at its infancy, and, when there were few fossil records. Thus, while the old ‘dispersal’ view might regard mangrove taxa originated in the IWP and subsequently dispersed to other parts of the world, a modern ‘vicariance’ view is that mangroves evolved around the Tethys Sea during the Late Cretaceous [6,15]. Phylogeographic analysis of one of the most widespread mangrove genera, *Rhizophora*, integrating genetic relationships, fossil records and geological/tectonic processes, will help to elucidate the role of vicariance and long-distance dispersal in the historical development of contemporary biogeographic patterns of mangroves, and to provide objective evaluation of these different but not mutually exclusive views. *Rhizophora* is a dominant genus of the most widespread mangrove family, the Rhizophoraceae. The genus is relatively old amongst cosmopolitan mangrove genera, and it has notable disjunct species distributions in both the AEP and IWP. Fossils of *Rhizophora* are recorded from the Palaeocene (55.8-65.5 Ma) onwards in major global regions [5]. All *Rhizophora* taxa are characterized by large water-buoyant propagules with a remarkable ability for long-distance dispersal [3]. For instance, recent genetic study using rapidly evolving microsatellites showed that *Rhizophora mangle* has dispersed over 3,000 miles from the north to south of the Brazilian coast since the end of the last glacial period [16]. While *Rhizophora* species are widespread in the world, only six (plus an equivalent number of hybrids) are described: *Rhizophora apiculata*, *Rhizophora mucronata*, and *Rhizophora stylosa* in the IWP; *Rhizophora mangle* and *Rhizophora racemosa* in the AEP; and *Rhizophora samoensis*, the only species found naturally in both regions [6]. In addition to the presence of major disjunctions in *Rhizophora* species distributions, the extant populations are not morphologically uniform and continuous at the intraspecific level [6], partly due to persistent introgressive hybridization, for example, among the New World *Rhizophora*[17]. While the reason for these disjunct occurrences might be complex, once created most discontinuities were persistent over millions of years – as evidenced by Wallace’s Line in the IWP region.

**Figure 1:**
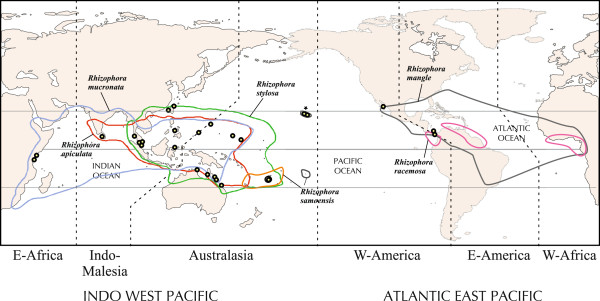
**Map showing distribution range of *Rhizophora *species in the Indo-West and Atlantic-East Pacific.** Dots indicate the collection sites included in this study and locality information are presented in Table 1. Asterisk indicates introduction occurrence.

Geographical disjunctions of plant and animal lineages can arise through historical long distance dispersal to new areas or through vicariant events that create physical barriers to gene flow and hence facilitate population divergence or allopatric speciation. Although the distributional patterns associated with vicariance may not be always distinct from those caused by dispersal, this can be tested by comparing selected scenarios of evolutionary relationships among disjunct and other lineages, molecular dating of lineage-splitting events, timing of geological processes or tectonic movements, as well as dispersal ability of the organisms of interest. There is also ample evidence for lineage diversification via a combination of mechanisms including both dispersal and vicariance [18]. The objectives of this study are to elucidate evolutionary relationships among its geographical lineages of *Rhizophora* (Figure 1; Table 1) in the light of the past geological events including continental drift, to illuminate major disjunctions at both inter- and intraspecific levels, and to generate a new hypothesis that combines both processes of vicariance and dispersal to explain the extant geographical distributions. Our estimation of divergence times for major lineages will shed light on the likely ancestral areas of *Rhizophora*.

**Table 1:** **Locality information of *Rhizophora *** **samples included in this study (see map in Figure **1**)**

**Taxon**	***N***	**Locality; Country**	**Site label**
*Rhizophora apiculata*			
	3	Cato River, Arnhem Bay; Australia	CAT
	5	Danitree River; Australia	DAI
	3	Embley River, Weipa; Australia	EMB
	3	Trinity Inlet, Carins; Australia	TRI
	3	Chuuk; Federated States of Micronesia	CHU
	2	Kosrae; Federated States of Micronesia	KOS
	3	Yap; Federated States of Micronesia	YAP
	2	Guam	GUA
	1	Iriomote Island; Japan	IRI
	3	North Sulawesi; Indonesia	IND
	3	Blue Lagoon, Cape Rachado; Malaysia	BLA
	2	Pulau Babi, TK Pelanduk; Malaysia	PBA
	2	Pulau Burong; Malaysia	PBU
	2	Sementa, Klang; Malaysia	SEM
	3	Phang Nga Bay, Phunket; Thailand	PNB
	3	Panay Island; Philippines	PHI
	3	West coast; Sri Lanka	SRI
*Rhizophora mangle*			
	3	Kahalu, eastern coast of Oahu; Hawaii, USA	KAH
	3	Waipahu, southern coast of Oahu; Hawaii, USA	WAI
	2	Atlantic coast, Panama	APA
	2	Pacific coast, Panama	PPA
	2	Pacific coast, Mexico	PMX
*Rhizophora mucronata*			
	3	Danitree River; Australia	DAI
	3	Trinity Inlet, Carins; Australia	TRI
	3	Gazi Bay; Kenya	GAZ
	3	Mida Creek; Kenya	MID
	2	Kosrae; Federated States of Micronesia	KOS
	3	Yap; Federated States of Micronesia	YAP
	2	Iriomote Island; Japan	IRI
	3	North Sulawesi; Indonesia	IND
	2	Sementa Klang; Malaysia	SEM
	3	Phang Nga Bay, Phunket; Thailand	PNB
	3	Panay Island; Philippines	PHI
	3	West coast; Sri Lanka	SRI
*Rhizophora racemosa*			
	3	Pacific coast, Panama	PPA
*Rhizophora samoensis*			
	3	VitiLevu Island; Fiji	VIT
*Rhizophora stylosa*			
	3	Cato River, Arnhem Bay; Australia	CAT
	4	Danitree River; Australia	DAI
	3	Embley River, Weipa; Australia	EMB
	3	Trinity Inlet, Carins; Australia	TRI
	2	Shoalwater Bay, Queensland; Australia	SWB
	3	Chuuk; Federated States of Micronesia	CHU
	3	Kosrae; Federated States of Micronesia	KOS
	3	Yap; Federated States of Micronesia	YAP
	3	Guam	GUA
	2	Iriomote Island; Japan	IRI
	3	North Sulawesi; Indonesia	IND
	2	Blue Lagoon, Cape Rachado; Malaysia	BLA
	3	Pulau Babi, TK Pelanduk; Malaysia	PBA
	3	Pulau Burong; Malaysia	PBU
	3	Panay Island; Philippines	PHI
	2	Taiwan	TAW
	3	Vitilevu Island; Fiji	VIT
*Bruguiera gymnorrhiza* (outgroup)			
	1	Vitilevu Island; Fiji	VIT
	1	Phang Nga Bay, Phunket; Thailand	PNB
	1	Pulau Burong; Malaysia	PBU

## Results

### Sequence variability among gene markers

Among the tested chloroplast regions in preliminary screening (Additional file 1: Table S1), introns of *nadh-A, ndhF, rpoC*, and *trnK* revealed the lowest amount of variation among taxa; all yielded less than 2% polymorphism. For the six intergenic spacer regions, *trnH-trnK* was the least variable followed by *rbcL-trnM* and *trnL-trnF*. Although *psbB-psbF* showed a sufficient amount of variation, the sequences were highly ambiguous due to substantial A/T repeats in this region. In contrast, the *trnH-rpl2* and *trnG-trnS* regions contain a high percentage of variable sites among the studied taxa and the sequences were shown to be accurate, without ambiguity. Together with the ribosomal ITS and multilocus ISSR markers, these regions provide adequate variation for resolving inter- and intraspecific relationships (Table 2).

**Table 2:** Sequence characteristics and models of DNA evolution selected by the Akaike Information Criterion (AIC) method implemented in jModeltest version 0.1.1

	**Chloroplast (CP)**		**Combined CP**	**Nuclear (NU)**	**CP + NU**
	***trn*** **G** ***-trn*** **S**	***trn*** **H-*rpl*** **2**		**Ribosomal ITS**	
**Total aligned length (bp)**	793	602	1395	656	2051
**Variable sites**	26	41	67	66	133
**Parsimony informative (PI) sites**	19	21	40	45	85
**Number of observed PI indels**	11	6	17	6	23
**Nucleotide substitutions per site**	0.02	0.02	-	0.02	-
**Nucleotide diversity**	0.04 ± 0.02	0.03 ± 0.02	-	0.02 ± 0.01	-
**Divergence range within** ***Rhizophora*** **(%)**	0.13-4.88	0.22-5.18	-	0.16-4.59	-
**Best-fit model of nucleotide substitution** (among the 88 tested models)	K81uf + I I = 0.82	TVM + I + G I = -0.67; G = 0.47	HKY + I + G I = 0.67; G = 0.8	GTR + G G = 0.40	GTR + I + G I = 0.67; G = 0.8

For the *trnH-rpl2* region, the total aligned length was 602 bp long with 21 parsimony informative sites and six indels. The average numbers of nucleotide substitution per site and nucleotide diversity of this region were 0.02 and 0.03 ± 0.02, respectively (Table 2). Two of the observed indels were shared by the Hawaiian and Atlantic Panama *R. mangle*. Four indels were found in *R. apiculata,* of which two were unique to the NW Pacific Islands and Australian populations. No length variation was found between *R. mucronata* and *R. stylosa* of the same geographical locations. Divergence values ranged from 0.22-5.18% among taxa. *Rhizophora samoensis* and *R. apiculata* showed the highest divergence among all pairs (3.78-5.18%) whereas *R. mucronata* and *R. stylosa* the lowest (0.22-2.22%).

For the *trnG-trnS* region, the total aligned length was 793 bp long with 26 parsimony informative sites and 11 indels. The average numbers of nucleotide substitution per site and nucleotide diversity of this region were comparable to *trnH-rpl2* (0.02 and 0.04 ± 0.02; Table 2). Six of the observed indels were shared by *R. mangle, R. racemosa,* and *R. samoensis.* Among them, two were unique to the Hawaiian and Atlantic Panama *R. mangle* and one was unique to *R. samoensis*. Three indels found in *R. apiculata* were shared between the NW Pacific Island and Australian populations. The remaining two indels were unique to the NW Pacific Island and Australian populations of *R. mucronata* and *R. stylosa*. Divergence values ranged from 0.13-4.88% among taxa, showing the highest between *R. mangle* and *R. mucronata*/*R. stylosa* (3.88-4.88%), and lowest between *R. mucronata* and *R. stylosa* (0.01-2.0%).

For the ITS region, the total aligned length was 656 bp long with 45 parsimony informative sites and 6 indels. The average number of nucleotide substitution per site and nucleotide diversity of this region were similar to those of *trnH-rpl2* and *trnG-trnS* (0.02 and 0.02 ± 0.01; Table 2). Four indels were found unique to *R. mangle, R. racemosa*, and *R. samoensis*, and one was unique to *R. samoensis*. A 14-bp insertion located in the ITS-2 region was shared by *R. apiculata*, *R. mucronata*, and *R. stylosa*. The ITS divergence values across taxa ranged from 0.16-4.59%, showing highest between *R. samoensis* and *R. mucronata*/*R. stylosa* (3.78-4.59%), and lowest between *R. mucronata* and *R. stylosa* (0.16-1.33%)*.* Within population divergence among individuals of the same species was less than 1% in the ITS sequences.

### Inter- and intraspecific relationships based on sequence and ISSR data

**Figure 2:**
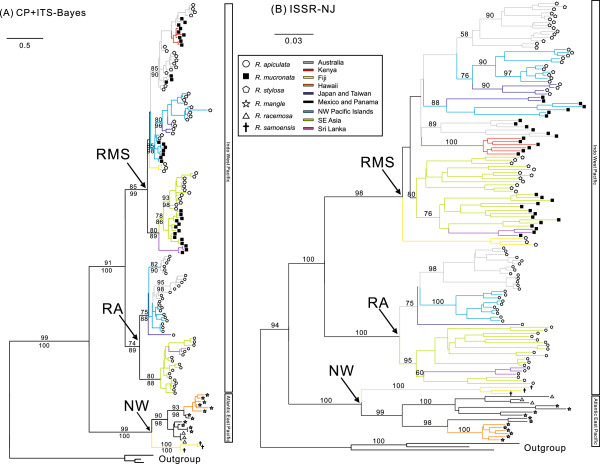
**Genetic relatedness among population samples of Rhizophora. (A)** Bayesian tree based on combined chloroplast and nuclear ribosomal ITS data using the GTR + I + G model (see Table 2 for details). Bootstrap (BS; above branch) and posterior probability (PP; below branch) values >50% are indicated. Individuals of *Bruguiera gymnorrhiza* were used for rooting purposes. **(B)** Neighbour-joining tree based on Jaccard distances, showing relatedness among population samples of *Rhizophora* species. Bootstrap values >50% are indicated.

The Incongruence Length Difference (ILD) test indicated no significant difference between the chloroplast and nuclear ribosomal ITS data (*P* > 0.05) and therefore the two datasets were combined to give a total-evidence phylogeny. The combined Bayesian phylogeny (Figure 2A) based on chloroplast and ITS data provided better resolution and stronger support to taxon relationships compared to separate sequence analyses (Additional file 2: Figure S2). *Rhizophora* taxa were divided into three strongly supported clades namely NW (BS 99%; PP 100%), RA (BS 74%; PP 89%), and RMS (BS 85%; PP 99%), corresponding to three groups of taxa. Clades RA and RMS are more closely related to each other (BS 91%; PP 100%) than to clade NW, and this topology reflects the deep divide between the IWP and AEP taxa. The relationships among *Rhizophora* species support those previously reported in Rhizophoraceae [19].

Clade NW contains *R. mangle* from Pacific and Atlantic Panama, Pacific Mexico, and Hawaii (introduced from Florida, USA), *R. racemosa* from Pacific Panama, and *R. samoensis* from Fiji. *Rhizophora mangle* of Atlantic Panama and Hawaii are grouped together (BS 93%; PP 98%), consistent with its human mediated introduction to Hawaii from the Atlantic source populations [20]. Fijian *R. samoensis*, despite being geographically closer to Australia and West Pacific islands, is sister to the AEP *R. mangle* and *R. racemosa*. Clade RA contains all individuals of *R. apiculata*. This clade is further divided into two subclades – one contains individuals from Australia, islands of the NW Pacific (Guam and Micronesia), and subtropical Asia (Japan; BS 75%; PP 88%); and the other contains individuals from Southeast Asia (Malaysia, North Sulawesi, Philippines, and Thailand) and Sri Lanka (BS 80%; PP 88%). Although relationships within the two *R. apiculata* subclades are unclear due to limited resolution, individuals from northern Australia (sites EMB and CAT) appear to be fairly different from eastern Australia (sites DAI, TRI, and SWB) based on length variation detected in the chloroplast sequences. Clade RMS contains all samples of *R. mucronata* and *R. stylosa*, whose individuals are not clearly distinguishable from one another but divided into two groups in accordance with geographical localities. Individuals from Southeast Asia and Sri Lanka are closely related (BS 80%; PP 89%; Figure 2A), forming a clade sister to individuals from Fiji, Australia, Kenya, islands of the NW Pacific, and subtropical Asia (Taiwan and Japan). *Rhizophora mucronata* from Kenya is nested within the clade containing Australian *R. mucronata* and *R. stylosa* (BS 85%; PP 90%), and this clade is shown to be sister to the NW Pacific *R. mucronata* and *R. stylosa* (BS 85%; PP 98%). The non-monophyly of *R. mucronata* and *R. stylosa* may relate to recent gene mixing and introgression events in local populations that merit in-depth evaluations of morphological features and population-level analyses. However, the taxonomic distinctiveness of the two taxa has no effect on inference of biogeographic relationships at a deeper time-scale.

Biogeographic relationships reflected from the NJ tree based on ISSR data (503 fragments from 145 individuals; Figure 2B) are consistent to those shown in the sequence-based phylogeny (Figure 2A). The AEP *R. mangle* and *R. racemosa* are closely related to each other (BS 99%) and they are sister to Fijian *R. samoensis* in clade NW (BS 100%). Within clades RA and RMS, individuals from Australia (sites CAT, DAI, EMB, SWB, and TRI), Kenya (sites GAZ and MID), NW Pacific (sites CHU, GUA, KOS, and YAP), and subtropical Asia (sites JAP and TAW) are separated from those from SE Asia (sites PBA, PHI, PNB, and IND) and Sri Lanka. Compared to sequence data, ISSR provides additional resolution to relationships among populations. For instance, *R. mucronata* and *R. stylosa* from Taiwan and Japan are shown to be closely related to individuals from NW Pacific (BS >75%). The northern Australian populations (sites CAT and EMB) are strongly associated with each other (BS ≥90%) and distinct from the eastern populations (sites DAI, SWB, and TRI).

### The timing of lineage divergence and ancestral areas reconstruction

**Figure 3:**
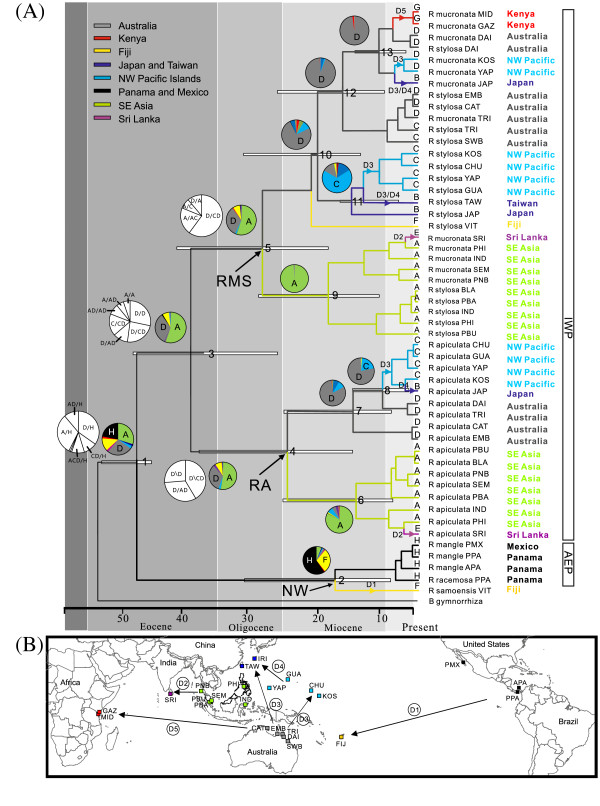
**Divergence time and dispersal routes of Rhizophora lineages. (A)** Chronogram of *Rhizophora* based on BEAST analyses of the combined chloroplast and ITS data. White bars indicate confidence interval of the estimated time of divergence of the respective nodes. Pie charts indicate the probable ancestral areas based on Lagrange (black and white) and Mesquite (color) analyses for the clade of interest. Relative probability and proportional likelihood values of ancestral distribution are presented in Additional file 3: Table S3. We presented only piechart for nodes where a variant or dispersal event was detected to identify the possible direction of the dispersal events based on the ancestral area inference. Arrows on branches as D1-D5 indicate potential dispersal events in the map below (see Results for details). **(B)** Map showing the sampling sites of *Rhizophora* in the Indo-West and Atlantic-East Pacific included in the present study (Table 1). The map image was prepared using MicroCAM v2.05 [21]. Distribution range of each species can be referred to Figure 1. Color label indicates the nine distribution areas defined according to past and present separation of major landmasses.

The split between the AEP and IWP *Rhizophora* was dated to approximately 47.6 ± 3.1 Ma (Figure 3A). In the AEP, the split between Fijian *R. samoensis* and American *R. mangle* and *R. racemosa* was estimated to be much more recent (~17.1 ± 10.3 Ma). The split between the Panama Pacific (plus Mexico Pacific) and Panama Atlantic lineages of *R. mangle* was dated to the Pliocene (~4.2 ± 7.4 Ma). In the IWP, the divergence of *R. apiculata* from *R. mucronata* and *R. stylosa* likely occurred much earlier during the Eocene (~38.9 ± 12Ma), although these species co-occur in many areas of the Indo-West Pacific and have a similar wide distribution range. In both clades of *R. apiculata* (RA) and *R. mucronata-R. stylosa* (RMS), the split between {Southeast Asia, Sri Lanka} and the {Australia, Kenya, Northwest Pacific Islands, subtropical Asia} lineages was dated to the Oligocene-Miocene boundary (~29-24 Ma). The divergence between the Kenyan and Australian lineages likely occurred in the Late Miocene (~6.9 ± 4.8 Ma).

Lagrange analyses indicated a relatively high dispersal rate (λ_D_) of lineages compared to the extinction rate (λ_E_) in *Rhizophora* (λ_D_ = 0.187, λ_E_ = 0.037; ln *L* = -111.8). The first split (node 1 in Figure 3A) was equally likely to occur between Central America (H) and Australia (D) (probability of D/H = 0.43) as well as between Central America (H) and Southeast Asia (A) (probability of A/H = 0.43). Mesquite analyses showed the most probable ancestral areas for *Rhizophora* are Southeast Asia (proportional likelihood = 0.30), Australia (proportional likelihood = 0.26), and Central America (proportional likelihood = 0.22) (node 1; Additional file 3: Table S3).

### Inference of major dispersal events

At least five independent dispersal events can be inferred from Figure 3A. First, ancestors of *Rhizophora* dispersed between Central America and Fiji (node 2 in Figure 3A; D1 in Figure 3B). Second, ancestors of *R. mucronata* dispersed between SE Asia and Sri Lanka (nodes 6 & 9 in Figure 3A; D2 in Figure 3B). Third, ancestors of *R. apiculata* and *R. mucronata*/*R. stylosa* dispersed between Australia and the western Pacific Islands (nodes 7, 11 & 12 in Figure 3A; D3 in Figure 3B). Fourth, the ancestors of *Rhizophora* dispersed between the western Pacific Islands and Taiwan and southern Japan (nodes 11 & 13 in Figure 3A; D4 in Figure 3B). And fifth, *R. mucronata* dispersed between Australia and East Africa crossing the Indian Ocean (node 13 in Figure 3A; D5 in Figure 3B).

## Discussion

### Historical vicariance and extinction

The deep divergence between IWP and AEP *Rhizophora* species groups could result from the following scenario: the ancestral Rhizophoras evolved and dispersed along the Tethys seaway westward into the Atlantic (along the Mediterranean and Arabian coasts through northern Spain and southern France to West Africa) and further into the east Pacific (long before the closure of the Panama Isthmus); and eastwardly split into Australia and Southeast Asia during the Late Cretaceous or early Eocene. The closure of the Tethys Seaway by the mid-Tertiary (34–50 Ma) created a physical barrier that completely terminated the AEP and IWP exchange route, and resulted in subsequent independent diversifications within the AEP and IWP regions. There is an abundant fossil pollen record of *Rhizophora* supports this scenario [22,23]. The earliest record of the *Rhizophora* type pollen is claimed to be from the Paleocene of Australia (~60 Ma) [24]. In Southeast Asia and South America, the occurrence of *Rhizophora* is dated to as early as the Upper Eocene (~50 Ma) [25-27]. There are documentations of a high percentage of *Rhizophora* pollen in the Oligocene of Puerto Rico [28] as well as from the Oligo-Miocene sediments of Mexico [29]. The presence of *Rhizophora* fossils in southern France (~50 Ma) [30] and in the London Clay (~45 Ma) [31] dated at the Mid-Eocene also provides evidence for ancient Tethyan dispersals. These abundant fossils present in the areas surrounding the ancient Tethys Sea suggest likely origination of the earliest mangroves in these locations. Because fossil deposits of *Rhizophora* in the Atlantic Caribbean region or West Africa are scarce, these areas are less likely to be the center of origin. Further phylogeographic studies on expanded mangrove samples are needed to test all alternative hypotheses on the origination of mangroves. The three ancestral areas – the Americas, Australia and SE Asia – identified in the present study likely reflect ancient Tethyan distribution and dispersals to these regions prior to the Oligocene, but not necessarily themselves being the natal areas where *Rhizophora* originated. *Rhizophora* in the primal area of origination could subsequently become extinct due to climate changes and historical vicariant events. The ancestral Rhizophoras that once existed along the Tethys Seaway, Mediterranean and Arabian coasts and Europe could have become extinct by the Cenozoic with its notable cooling and drastic environmental changes during the late Tertiary [32,33]. In addition, when continental fragments of Gondwana, such as India, Arabia, and Apulia collided with the rest of Eurasia, this might have impacted the old shorelines along with the ancestral mangrove habitats and created new barriers to dispersal.

The historical dynamics between vicariance and dispersal would undoubtedly have impacted present-day mangrove distributions. Also, probabilities of local extinctions could greatly differ between geographical regions as species expand and contract in their distributions in response to climate changes. For example, Pleistocene glacial events affected global climate and sea levels, which likely modified present mangrove distributions in different regions of the world [34]. During this period of maximum glaciation, the drop in sea level created land connections and hence opened more suitable habitats for mangrove expansion among the Malay Peninsula, Sumatra, Borneo, and Java, as well as between Australia and Papua New Guinea, whereas arid climates might have contributed to range contraction of mangroves in Africa and the New World [35]. Although the historical pattern of mangrove expansions and contractions, as well as local extinctions and recolonizations, is still largely unknown, the surviving lineages certainly represent only a fraction of the total evolutionary diversification. Because our samples could not possibly include the extinct ancestral populations from the site of origination, and also because the current oldest fossil pollen could be much younger than the actual age of those extinct ancestors, the present estimates of divergence time among extant lineages based on existing molecular variation in the sampled populations could be taken as conservative estimates of the actual ages of *Rhizophora* diversifications. Thus the actual divergence time between IWP and AEP Rhizophoras could be older than the present estimate (Figure 3).

Further down to the south, Wallace’s Line marks a major, well-known discontinuity along the island archipelago from SE Asia to Australasia. This feature was consistent with our observation of a deep divergence between Southeast Asia (including Malaysia, Thailand, North Sulawesi, and Philippines) and the Australian-west Pacific populations (Figure 3A). Given their present-day geographical proximity, this diversification would be inexplicable if not in the light of historical vicariant events and ocean current directions or circulation patterns in these regions. The northward movement of the Australia plate following its breakup from Antarctica would facilitate ancient dispersals to its seashores, as evidenced by fossil pollen dated to about 60Ma [24] and macro fossils of later times [15] As the Australian plate reached its current situation in close proximity to SE Asian populations, it would have created opportunities for more frequent exchanges of propagules among their populations. However, the genetic discontinuity observed between the SE Asian and Australian *Rhizophora*, which has been maintained over millions of years, suggests the long-term preservation of established ancestral gene pools. The directions of local ocean currents might have played a major role in separating present-day Australian Rhizophoras from SE Asian populations. As vicariant events are expected to affect co-distributed taxa simultaneously, similar patterns of phylogeographic disjunction are found in both *R. apiculata* and *R. mucronata/stylosa* within the IWP. These patterns are also seen in morphological characters among populations of *R. apiculata*, with one form existing in Australia while a different form occurs throughout SE Asia [6]. Furthermore, a similar phylogeographic pattern exists in the mangrove genus *Bruguiera*[36]. Individuals from northern Sulawesi of Indonesia were genetically similar to those of Hainan Island but differentiated from the Australian populations of both *B. gymnorrhiza* and *B. sexangula* – a pattern consistent with the present findings for *Rhizophora*.

### Major long-distance dispersal events

The IWP’s richer biodiversity and distinctive distributional disjunctions are generally accepted as being consistent with a more complex geological history of tectonic movements in the region compared to the AEP, but there have been differing views on the effectiveness of long distance dispersal influencing the distributions of mangrove species [37]. *Rhizophora* propagules reportedly survive well at sea, and they are known to successfully travel longer distances than other mangrove species like *Bruguiera, Avicennia*, and *Sonneratia*[3,4,38]. In addition, lower sea levels during the Eocene might also have enhanced the dispersal across narrowed ocean expanses and via additional islands [5,15]. The present study used evidence of relationships between extant populations to provide information on the effectiveness of long distance dispersal on the distribution of *Rhizophora* entities.

### Trans-Pacific dispersal from the AEP to the IWP

The monophyly of the American and Southwest Pacific species (*R. mangle, R. samoensis* and *R. racemosa*) revealed a transpacific dispersal, likely from the AEP to IWP in the Miocene and thus unrelated to possible human activities. *Rhizophora mangle* and *R. racemosa* are common in western Atlantic mangroves, whereas *R. samoensis* occurs both in the eastern Pacific and its disjunct range in the IWP [39]. The recognition of Central America as one of the ancestral areas of *Rhizophora* coincides with the occurrence of *R. racemosa* and *R. mangle* type pollen in Mexico during the Oligocene-Miocene period [22,28,29]. However, we cannot rule out the possibility that these fossil pollen could be relicts of an extinct lineage originated elsewhere, although the continuous fossil record in areas surrounding the ancient Tethys Sea supports the expansion of ancestral Rhizophoras into the AEP through the Tethys. Morphologically, a single and common origin for *R. mangle, R. racemosa,* and *R. samoensis* was supported also by their shared characters: blunt and recurved leaf margins, long peduncles, 2–5 flowers per inflorescence, and waxy-yellow mature flower buds [6]. While our data provide genetic evidence for the shared ancestry of the Fijian *R. samoensis* and American Rhizophoras, there remains unanswered questions about how this species crossed more than 8,000km of the southern Pacific Ocean, especially where many islands in between have apparently suitable habitat, and yet unoccupied. By contrast, the American continental landmass constitutes strong geographical barriers to dispersal of red mangroves in the AEP, splitting the Pacific and Atlantic populations of *R. mangle* and *R. racemosa* into distinct genealogical units [40,41].

*Rhizophora samoensis* in the SW Pacific is well established in numerous islands, spread across five island states [42], including Samoa, Tonga, Fiji, Vanuatu and New Caledonia. Its apparent long establishment there is affirmed by occurrences in every estuary around the large island of New Caledonia, which is positioned at the western limit of the species. This oddly contrasts with the comparatively limited distribution of *R. apiculata* on the island to only a few estuaries with higher rainfall in the north. Furthermore, *R. samoensis* is the only *Rhizophora* present in Samoa – the eastern limit of this SW Pacific range. These observations support a prehistoric trans-Pacific dispersal of *R. samoensis* to the SW Pacific islands from the AEP [40], whereas *R. stylosa* and *R. apiculata* reached these islands from the opposite direction.

It is cogent to ask why a genus with such a capacity for long distance dispersal, should be so restricted in the South West Pacific. What is stopping *R. samoensis* from extending further west to Australia or New Guinea? And, why have *R. stylosa* and *R. apiculata* not dispersed further eastward in the Pacific, let alone to the AEP? There are apparently other factors that must be taken into account to address these questions, such as the direction of ocean current, wind, water temperature, rainfall, as well as suitable habitat for subsequent establishment following each dispersal event. While detailed information on paleocean currents in the Pacific is lacking and historical circulation pattern could be more complex and different from the present, global warming in the earliest Eocene might have contributed to large-scale changes in deep-ocean circulation and determined much of today’s major ocean current system [43-45]; Additional file 4: Table S4. Ocean currents have been shown to play a key role in shaping the distribution and connectivity of marine organisms [46-51]. For instances, the North Equatorial Current, which situates at 2°N and shifts direction four times a year (January-February and August-October flows are eastward; November-December and March-April flows are westward) [52], has been shown to carry planktonic larvae [53], sea basses [54], and coral reef fishes [55] from the eastern Pacific toward the IWP. This, together with the westward flowing South Equatorial Current, which situates between 1°N and 3S-5S [56], could have provided a route of *Rhizophora* dispersal from the East Pacific to Fiji. Despite the fact that eastward flow of Antarctic Circumpolar Current and Tasman Current brought dense Antarctic waters from the southwest to eastern Pacific since the late Eocene and early Oligocene [43,57,58], these waters might be too cold for any *Rhizophora* propagules to survive over large stretches of the open ocean. Also, the closure of the Central American Isthmus (Pliocene-3Ma) could have weakened the western flow of the Equatorial Countercurrent [45,59]. Thus, where discontinuities have been maintained over millions of years, direction of ocean currents and water temperatures may have prevented eastward dispersal of *Rhizophora* propagules from the IWP into the AEP. As geographical and climatic circumstances are not constant, dispersal events must be episodic with chance establishment.

### Transoceanic dispersals in the IWP

Our data revealed at least three major transoceanic dispersals within the IWP, including Southeast Asia – Sri Lanka; Australia – NW Pacific; and Australia – East Africa. Genetic associations amongst *R. apiculata* and *R. mucronata* in Southeast Asia and Sri Lanka suggest the dispersal of *Rhizophora* propagules from the SE Asian lineages to Sri Lanka across the Bay of Bengal (Figure 3B) [60]. The founding event may have occurred as early as the Late Miocene when the Indian landmass was in closest proximity to Southeast Asia after its separation from the Gondwana supercontinent. Prior to Miocene, water possibly flowed west from the Pacific into the Indian Ocean by the Equatorial Current. The Indonesian Passage was viewed as the last of the Tethyan ocean gateways. This deepwater passage through the Indonesian Archipelago became severely restricted in the middle Miocene by the effect of tectonic reconstructions [61,62], through which there is still significant flow from the Pacific to the Indian Ocean [63-65]. The differentiation of radiolarian faunas between the Indian Ocean and western Pacific at about 11 Ma is one example suggesting some restriction of water exchange through the Indonesian Passage to develop different water masses on either side of the Indonesian Gateway [66,67]. However, Leinen [68] and Keller [69] proposed that the Equatorial Undercurrent initiated by circa 11 Ma in the equatorial Pacific could have permitted a westward flow of the Pacific water into the Indian Ocean subsequent to the partial closure of the Indonesian Seaway. This is exemplified by the wide of geographical distribution of *Hibiscus tiliaceus*, a semi-mangrove with water-buoyant and salt-tolerant seeds, which has dispersed long distance via ocean current throughout the northwestern Pacific Ocean and the Indian Ocean [70].

For Australian – NW Pacific lineages (from Guam, Yap, Chuuk, Kosrae, Taiwan, and Iriomote), there may have been an island-hopping radiation of *Rhizophora* species from Australia northward into the Pacific. Many plant species are shown to be similarly dispersive across the Pacific Ocean [71,72]. Moreover, major tectonic events in the Cenozoic period involving the collision of Australian and Pacific Plates, gave rise to chains of islands probably within the last 40 million years [73]. For instance, Guam of the Mariana Islands, one of the oldest (45–40 Ma) was formed during the Eocene [74,75]. Similarly, in the Caroline Islands, Yap and Chuuk were formed during the Miocene, while Kosrae, the most easterly of these islands, formed in the Late Pliocene (1.4-2.6 Ma) [76]. Also during the Miocene, Iriomote (southern Ryukyu Islands of Japan) and the islands of Taiwan were established [77,78]. Mangroves on these islands appear associated with the Australian lineages, and possibly moved northward following a clockwise ocean circulation. Dispersal of Pacific island derivatives further north to Taiwan and southern Japan may also have been facilitated by ocean currents [74,75,79].

A third transoceanic dispersal is revealed by the close relationship among *R. mucronata* of Kenyan and Australian populations. As *R. mucronata* from Kenya is nested within the Australian clade, this implies the direction of dispersal is from Australia to East Africa across the Indian Ocean, likely facilitated by the south equatorial current. Previously, the Indian Ocean has been considered as an effective historical and present-day barrier to dispersal, on the basis of species composition in East Africa (a subset of the highly diverse mangroves in the East Indian Ocean and beyond) [37], as well as genetic evidence on *Avicennia marina* across its range – the presence of a high number of private SSR alleles in each of the distant populations from South Africa, United Arabic Emirates, India and the Malaysian-Australasian region [80]. Compared to *Rhizophora*, the much smaller *Avicennia* propagules render the species the poorer long distance disperser, despite its common occurrence on both east and west Indian Ocean shorelines. On the other hand, a much larger sample sizes are needed to accurately capture the allelic compositions at highly polymorphic SSR loci. Recent studies of its congener *Avicennia germinans* in the AEP have provided supporting evidence for long-distance oceanic dispersals based on a close genetic relationship between populations from West Africa and South America [38]. The trans-Atlantic dispersals were considered to be relatively recent and governed by the strength and direction of the equatorial Atlantic Ocean current during the Quaternary.

## Conclusions

Our comprehensive biogeographic study of the most representative mangrove genus *Rhizophora* sheds light on the patterns of regional associations of the genus and timing of lineage divergence, allowing the inference of a global history of its evolutionary diversifications. This study presents new evidence of phylogeographic patterns of *Rhizophora* across its global range. We postulate the hypothesis that combines both historical vicariance and oceanic long-distance dispersal to account for mangroves’ modern geographical distributions, the observed major disjunctions and key phylogenetic affinities. Based on our findings, including a deeply divergent monophyletic AEP clade, it is most likely that the primal ancestors of *Rhizophora* originated in the shore of the ancient Tethys Sea during the Cretaceous. Their subsequent dispersal along the Tethys Seaway was followed by notable instances of vicariance that divided the global regions before further independent diversifications in the IWP and AEP. Within each region, especially in the IWP, there were independent long histories of tectonic movements and multiple, relatively more recent, long-distance dispersals. It must be noted though that the present conclusions, which partly rely on our current knowledge of early fossil distributions along the Tethys Sea and in the inferred ancestral areas, are tentative and may subject to modification if there are new discoveries of abundant fossil deposits from other potential areas of origin, as well as more complete sample coverage in population localities and genomic markers.

## Methods

### Taxon sampling

To maximize geographical coverage of *Rhizophora*, we included 145 individuals collected from 26 localities in the IWP and AEP regions, representing all the six species: *R. apiculata, R. mangle, R. mucronata, R. racemosa, R. samoensis,* and *R. stylosa* (Figure 1, Table 1). Hybrids of *Rhizophora* (specifically *R. ×lamarckii*, *R. ×selala*, and *R. ×annamalayana*) are not included in this study because these hybrids have been shown to be *F*1s [81] and are sterile or have much reduced fertility [6,42]. Inclusion of hybrid individuals can create conflict between chloroplast and nuclear trees [81], and obstruct our goal of inferring biogeographic history of the genus. Individuals of *Bruguiera gymnorrhiza*, from the sister genus to *Rhizophora*, were used as outgroup to root phylogenetic trees. Genomic DNA was extracted from the silica gel dried leaf tissues using DNeasy Plant Mini Kit (QIAGEN) following the manufacturer’s protocol.

### Molecular markers

In our initial primer screening, ten coding and non-coding cpDNA regions were amplified and sequenced using a subset of individuals representing five of the *Rhizophora* species (Additional file 1: Table S1). We estimated the percentage polymorphism of each region by dividing the number of variable sites by the total surveyed length. Regions of 5-15% polymorphism should be sufficiently informative for studying taxon relationships [82]. Therefore, we selected the two highly polymorphic chloroplast intergenic regions, *trnH-rpl2* and *trnS-trnG*, in addition to nuclear ribosomal internal transcribed spacer (ITS) for the present study using the published primers [83,84]. Although the *psb*B-*psb*F region was also sufficiently polymorphic (5.24% polymorphism; Additional file 1: Table S1), this region was not selected for use because it contains an exceptionally high A/T repeats that resulted in ambiguous sequences. All PCR amplifications of our selected regions yielded single and sharp bands and the purified products were sequenced directly on an ABI 3100 (Applied Biosystems) automated DNA sequencer with the BigDye terminator cycle sequencing kits. In addition to the chloroplast and ITS sequences, multilocus marker Inter-Simple Sequence Repeats (ISSRs) was used to resolve relationships particularly at the intraspecific levels and to compare with the sequence-based results. Among 100 ISSR primers (UBC set no. 9), 16 were selected based on two criteria – fragment reproducibility and variability among and within species (Additional file 5: Table S5).

Amplification was conducted in a 20 ul reaction mixture containing 10-20 ng of genomic DNA, 2 ul 10 × PCR buffer (10 mM Tris–HCl, 50 mM KCL, 0.1% Triton × 100), 2.5 mM MgCl_2_, 1.5U Taq polymerase, 0.2 mM dNTP, and 0.3 uM primer. Reaction was performed in MJ Researcher PTC-100TM programmable thermal controller, with an initial denaturation at 94°C for 5 min, followed by 35 cycles at 94°C for 30 sec, 49°C for 45 sec, and 72°C for 1 min 30 sec, with a final 7 min extension at 72°C. The amplified products were resolved electrophoretically on a 2% agarose gel in 0.5 × Tris-borate (TBE) buffer and visualized under UV light. The amplified ISSR fragments were scored as either presence (1) or absence (0) for each individual to generate a binary data matrix. The individuals used in the ISSR assay were identical to those used in the sequence analyses. They were drawn from a larger sample collected from each locality being used in our ongoing investigation of population genetic structure.

### Data analyses

All sequences were aligned with MUSCLE version 4.0 [85] and manually adjusted with the Sequence Alignment Editor version 1.d1 [86]. Gaps that are parsimony informative were coded into multistate characters with SeqState version 1.32 [87] and appended to the sequence matrices. Phylogenetic analyses were conducted using the maximum likelihood (ML) criterion in PAUP* 4.0b10 [88] and the Bayesian criterion using MrBayes version 3.0b4 [89]. The nucleotide substitution models of the chloroplast and ITS data were determined by the Akaike Information Criterion (AIC) method implemented in jModeltest version 0.1.1 [90]. The GTR (General Time Reversible) + G (gamma) model was chosen for the ITS data, the HKY + G + I (proportion of invariable sites) model for the chloroplast data, and the GTR + I + G model for the combined chloroplast and nuclear ITS data. These best-fitting models and related parameters were used in the ML and Bayesian analyses. For ML analyses, all searches were heuristic with TBR branch swapping. Bootstrap support (BS) was assessed with 1,000 pseudo-replicates. Bayesian analyses were performed with four Markov chains each initiated with a random tree and with two independent runs for 50,000,000 generations each (until posterior probabilities and other parameters were converged), sampling every 1000^th^ generation. Likelihood values were monitored for stationarity with Tracer v1.4.1 [91]. Trees and other sampling points prior to the burn-in cut-off (i.e. approximately a quarter of the total sampling points when stationarity was reached) were discarded and the remaining trees were imported into Phyutility v2.2 [92] to generate a majority-rule consensus. Posterior probability (PP) values were used to evaluate node support in the Bayesian trees (TreeBase accession number 15468).

Compatibility of tree topologies and bootstrap values were used for initial visual assessments of congruence between datasets. All chloroplast sequences were combined in phylogenetic analyses because these regions are linked as a single unit and no well-supported conflict is detected among individual trees. To test for the significance of congruence between the chloroplast and nuclear datasets, Incongruence Length Differences tests (ILD) [93] as implemented in PAUP* (‘partition homogeneity test’ option) were conducted. We ran 1000 homogeneity replicates each with 10 random sequence additions using parsimony heuristic searches with tree bisection and reconnection (TBR) branch swapping and ACCTRAN optimization options. The statistical significance of incongruence was assessed by two non-parametric tests in PAUP* [88].

For the binary ISSR data, pairwise genetic distances between individuals were obtained by computing the Jaccard coefficient (JC), which does not consider the shared absence of a band between individuals as similarity [94]. Neighbour-joining (NJ) tree based on the JC distance matrix was constructed using PHYLIP version 3.66 [95].

### Divergence time estimation

We estimated divergence times among *Rhizophora* lineages using a fossil-calibrated relaxed molecular clock with BEAST v1.5.3 [96] based on the combined chloroplast and ITS data, as no significant conflict was detected between the two data sets. Outgroup *Bruguiera* was constrained to be sister to all *Rhizophora* taxa based on the known relationships between the two, but other relationships were unconstrained. An uncorrelated lognormal (UCLN) relaxed-clock model was applied to allow rate variation/independence across branches. A Yule tree prior that assumes a constant lineage birth rate for each branch in the tree was specified to model speciation. Two independent MCMC runs were performed for 50,000,000 generations, sampling every 1000^th^ generation. The GTR + G and HKY + G + I models were used, respectively, for the ITS and chloroplast data. Posterior probabilities and other parameters were shown to converge after 50 million generations. Likelihood values were monitored for stationarity, and trees and other sampling points prior to the burn-in cut-off (12,500 out of 50,000 trees) were discarded. The known fossil record suggests that species associated with mangroves evolved soon after the appearance of flowering plants, with the earliest records of *Nypa* (a mangrove palm) placed in the Upper Cretaceous-Paleocene times (69 Ma) [97,98]. Assuming that the age of the crown Rhizophoraceae (*Bruguiera* and *Rhizophora*) is not older than the earliest records of mangrove plants (~69 Ma) but could be older than the earliest *Rhizophora* fossil pollen (~60 Ma) [24], we modeled the root node using a lognormal prior distribution with offset value of 60, mean of 1.5, and standard deviation of 0.5 to encapsulate the upper Paleocene in the prior of divergence time estimation.

### Ancestral area reconstruction

The maximum likelihood (ML) method implemented in Lagrange v2.0.1 [99] was used to infer ancestral areas of *Rhizophora* lineages. The consensus Bayesian tree generated from the combined cpDNA and ITS data was used as the primary input together with the distribution matrix of taxa. Eight distribution areas were defined based on past and present separation of major landmasses (Figure 1). They include A–Southeast Asia; B–Subtropical Asia including Japan and Taiwan; C–Islands of northwest Pacific (Guam and Micronesia); D–Australia; E–Sri Lanka; F–Fiji; G–East Africa (Kenya); and H–Central America (Atlantic and Pacific coast of Panama and Pacific coast of Mexico). Because mangroves in Hawaii were not present until introductions in the 1920s [20], this area was excluded in the ancestral area inference*.* We treated the Atlantic and Pacific American/Mexican coasts as one area (H–Central America) based on the likelihood that Rhizophoras from these mangrove areas share a common source origin and that the split should be relatively recent given the Central American seaway was only almost completely closed at 3.6-3.8 Ma [100], although some records indicated the last breach of the Panama Isthmus occurred as late as 1.9 Ma [101]. Based on geological history and tectonic events, some of the pre-defined areas such as islands of northwest Pacific (C) and Fiji (F) are considered too young to be the ancestral areas of *Rhizophora.* Therefore, the analyses were conducted with the following 35 possible ancestral ranges: AB, AD, AE, AG, AH, BD, BE, BG, BH, DE, DG, DH, EG, EH, GH, ABH, ADH, AEH, AGH, ABD, ABE, ABG, ADE, ADG, AEG, BDE, BDG, BDH, BEG, BEH, BGH, DEG, DEH, DGH, and EGH.

In addition to Lagrange, we used the ML approach implemented in Mesquite v2.01 [102] to estimate the most probable ancestral area(s) of nodes where a lineage-splitting event was inferred. The Mk1 (Markov k-state 1 parameter) model that gives equal probability (or rate) for changes between any two-character states (i.e. areas in this case) was applied. The proportional likelihood values of all estimated areas for each node were also estimated.

## Competing interest

The authors declare no competing interests.

## Authors’ contributions

EL performed molecular experiments, data analyses, and wrote the manuscript. ND involved in the design of the study and sample collection. MS conceived and designed the study and wrote the manuscript. All authors read and approved the final manuscript.

## Supplementary Material

Additional file 1 **Information of the 10 coding and non-coding chloroplast regions and nuclear ribosomal ITS tested in preliminary screening using a subset of *Rhizophora *** **samples.** Click here for file

Additional file 2 **Bayesian trees based on (A) chloroplast and (B)nuclear ribosomal ITS data.** Bootstrap (BS; above branch) and posterior probability (PP; below branch) values >50% are indicated. Individuals of *Bruguiera gymnorrhiza* were used for rooting purposes. Click here for file

Additional file 3 **Comparisons of ancestral areas reconstructions by Lagrange and MK-1 tests implemented in Mesquite.** Areas are defined as follow: A–Southeast Asia; B–Japan/Taiwan; C–Northwest Pacific Islands; D–Australia; E–Sri Lanka; F–Fiji; G–Kenya (East Africa); H–Central America. Proportional likelihood values of >0.2 are highlighted in bold to indicate the most likely ancestral area(s) identified by the MK-1 test. ^a^Node numbers correspond to those in Figure 3A. Click here for file

Additional file 4 **GenBank accession number of *Rhizophora *samples included in this study.** For each gene region, given sequence divergence among individuals of the same species from the same locality was less than 1%, one sequence was selected to represent a taxon from each locality. Asterisks denoted individuals used in Lo (2010). Alignment and tree were deposited in TreeBase (accession number 15468). Click here for file

Additional file 5 Sequences of the 16 primers used for PCR amplification of inter-simple sequence repeat (ISSR). Click here for file
