# Supplementary material for: Phylogeographic pattern of Rhizophora (Rhizophoraceae) reveals the importance of both vicariance and long-distance oceanic dispersal to modern mangrove distribution
Source: BMC Evol Biol. 2014 Apr 17;14:83. doi: 10.1186/1471-2148-14-83 (PMC4021169; doi:10.1186/1471-2148-14-83)
Supplement: Additional file 1 — Information of the 10 coding and non-coding chloroplast regions and nuclear ribosomal ITS tested in preliminary screening using a subset of Rhizophora samples. [file 1471-2148-14-83-S1.docx]

**Additional file** **1**. Information of the ten coding and non-coding chloroplast regions and nuclear ribosomal ITS amplified in a subset of individuals representing *Rhizophora* species. Percentage polymorphism is calculated based on the total number of variable sites divided by the surveyed length. Regions in bold were used for phylogenetic reconstructions of expanded samples in this study.

| **Region** | **Primer sequence (5’ to 3’)** | **Aligned length (bp)** | | **Polymorphism (%)** | **Reference** |
| --- | --- | --- | --- | --- | --- |
| **Chloroplast** |  |  |  | |  |
| *nadh-*A | F: TAT CTC TAC GTG CGA TTC G  R: ATC AC TAT ATC AAC TGT ACT T | 830 | <1 | | Olmstead et al. (1994) |
| *ndh*F | F: CTA TGG TAG CGG CGG GAA TTT TTC  R: CGA TTA TAT GAC CAA TCA TAT A | 989 | 0.68 | | Olmstead et al. (1994) |
| *rpo*C | F: TCG ATT GAA ACG AGT ACG ACC  R: CAC TGG AGG GCC AAT ACC TA | 800 | <1 | | Parducci et al. (1999) |
| *trn*K | F: GGG TTG CCC GGG ACT CGA AC  R: CAA CGG TAG AGT ACT CGG CTT TT | 1003 | 1.66 | | Dumolin et al. (1997) |
| *psbB-psbF* | F: GTT TAC TTT TGG GCA TGC TTC G  R: CGC AGT TCG TCT TGG ACC AG | 710 | 5.24 | | Hamilton et al. (1998) |
| *rbc*L*-trn*M | F: GCT TTA GTC TCT CTG TTT GTG G  R: TGC TTT CAT ACG GCG GGA GT | 650 | 2.6 | | Dumolin et al. (1997) |
| *trnH-trnK* | F: ACG GGA ATT GAA CCC GCG CA  R: CCG ACT AGT TCC GGG TTC GA | 820 | <1 | | Dumolin et al. (1997) |
| ***trnH-rpl*2** | F: CGG ATG TAG CCA AGT GGA TC  R: GAT AAT TTG ATT CTTT CGT CGC C | 602 | 6.85 | | Vaillancourt et al. (2000) |
| ***trnG-trnS*** | F: GCC GCT TTA GTC CAC TCA GC  R: GAA CGA ATC ACA CTT TTA CCA C | 793 | 5.86 | | Hamilton et al. (1998) |
| *trnL-trnF* | F: CGA AAT CGG TAG ACG CTA CG  R: ATT TGA ACT GGT GAC ACG AG | 734 | 2.52 | | Taberlet et al. (1995) |
| **Nuclear** |  |  |  | |  |
| Ribosomal ITS | F: TCC TCC GCT TAT TGA TAT GC  R: GGA AGT AAA AGT CGT AAC AAG G | 656 | 8.54 | | White et al. (1990) |
