# Supplementary figures and images for: Phylogeographic pattern of Rhizophora (Rhizophoraceae) reveals the importance of both vicariance and long-distance oceanic dispersal to modern mangrove distribution
Source: BMC Evol Biol. 2014 Apr 17;14:83. doi: 10.1186/1471-2148-14-83 (PMC4021169; doi:10.1186/1471-2148-14-83)

(A) CP

(B) ITS

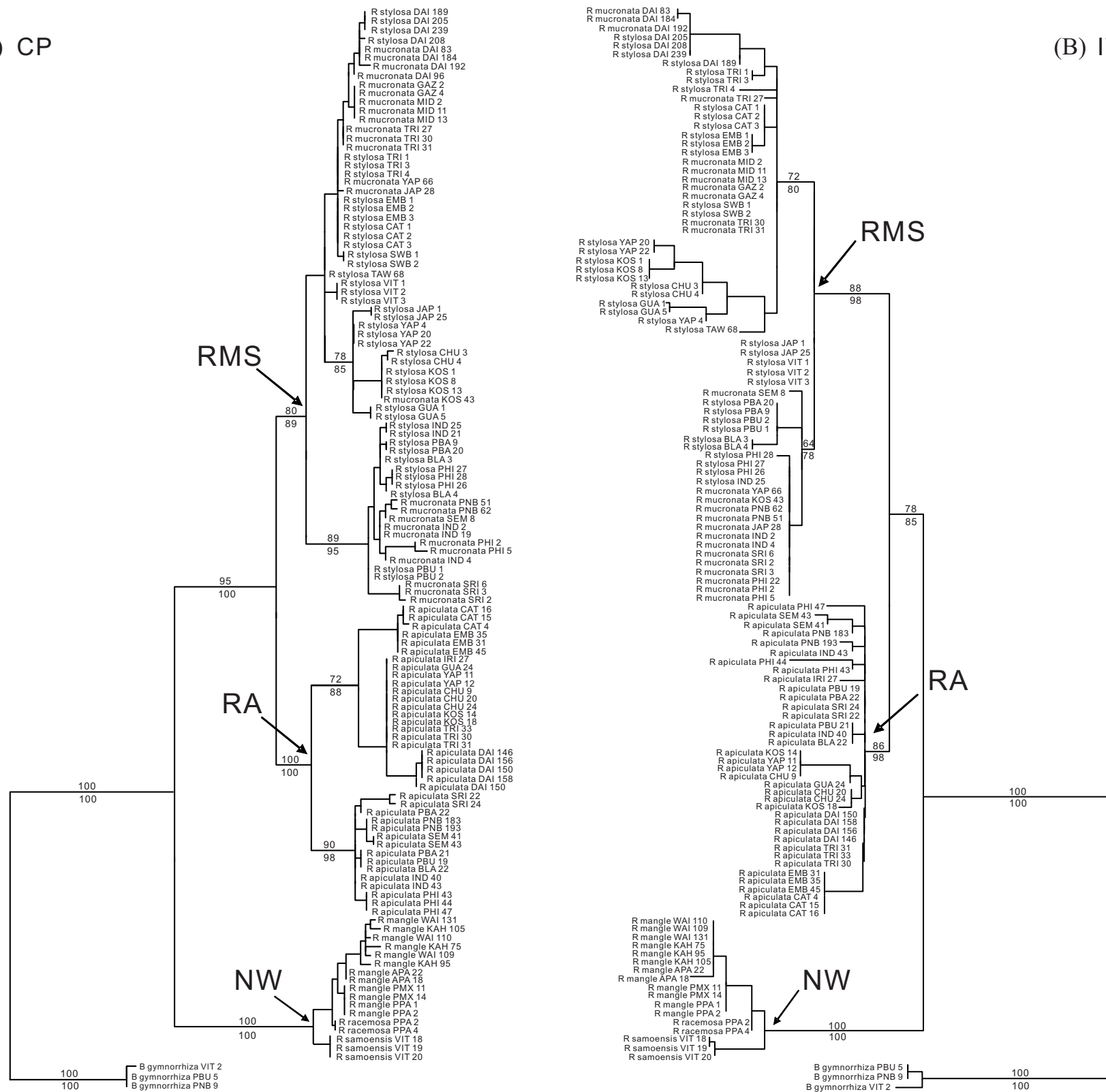

Supplement: Additional file 2 — Bayesian trees based on (A) chloroplast and (B)nuclear ribosomal ITS data. Bootstrap (BS; above branch) and posterior probability (PP; below branch) values >50% are indicated. Individuals of Bruguiera gymnorrhiza were used for rooting purposes. [file 1471-2148-14-83-S2.pdf]
