# Supplementary material for: Phylogeographic pattern of Rhizophora (Rhizophoraceae) reveals the importance of both vicariance and long-distance oceanic dispersal to modern mangrove distribution
Source: BMC Evol Biol. 2014 Apr 17;14:83. doi: 10.1186/1471-2148-14-83 (PMC4021169; doi:10.1186/1471-2148-14-83)
Supplement: Additional file 3 — Comparisons of ancestral areas reconstructions by Lagrange and MK-1 tests implemented in Mesquite. Areas are defined as follow: A–Southeast Asia; B–Japan/Taiwan; C–Northwest Pacific Islands; D–Australia; E–Sri Lanka; F–Fiji; G–Kenya (East Africa); H–Central America. Proportional likelihood values of >0.2 are highlighted in bold to indicate the most likely ancestral area(s) identified by the MK-1 test. aNode numbers correspond to those in Figure 3A. [file 1471-2148-14-83-S3.doc]

**Additional file** **4.** Comparisons of ancestral areas reconstructions by Lagrange and MK-1 tests implemented in Mesquite. Areas are defined as follow: A—Southeast Asia; B—Japan/Taiwan; C—Northwest Pacific Islands; D—Australia; E—Sri Lanka; F—Fiji; G—Kenya (East Africa); H—Panama and Mexico. Node number can be referred to the chronogram in Figure 3. Proportional likelihood values of >0.2 are highlighted in bold to indicate the most likely ancestral area(s) identified by the MK-1 test.

|  | Lagrange | | MK-1 test in Mesquite | | | | | | | | |
| --- | --- | --- | --- | --- | --- | --- | --- | --- | --- | --- | --- |
|  |  |  | Proportional likelihood values | | | | | | | | |
| Node | Ancestral range subdivision | Relative probability | A | B | C | D | E | F | G | H |  |
| 1 | D/H | 0.43 | **0.30** | 0.02 | 0.03 | **0.26** | 0.02 | 0.13 | 0.02 | **0.22** |  |
|  | A/H | 0.43 |  |  |  |  |  |  |  |  |  |
|  | AD/H | 0.06 |  |  |  |  |  |  |  |  |  |
|  | CD/H | 0.05 |  |  |  |  |  |  |  |  |  |
|  | ACD/H | 0.03 |  |  |  |  |  |  |  |  |  |
|  | A/FH | 0.01 |  |  |  |  |  |  |  |  |  |
|  | D/FH | 0.01 |  |  |  |  |  |  |  |  |  |
| 2 | H/F | 0.33 | 0.06 | 0.01 | 0.01 | 0.02 | 0.01 | **0.28** | 0.01 | **0.59** |  |
|  | H/H | 0.34 |  |  |  |  |  |  |  |  |  |
| 3 | D/DC | 0.64 | **0.55** | 0.01 | 0.02 | **0.33** | 0.01 | 0.07 | 0.01 | 0.01 |  |
|  | A/AC | 0.23 |  |  |  |  |  |  |  |  |  |
|  | A/AD | 0.11 |  |  |  |  |  |  |  |  |  |
|  | A/DH | 0.11 |  |  |  |  |  |  |  |  |  |
| 4 | D/AD | 0.39 | **0.67** | 0.01 | 0.03 | **0.22** | 0.03 | 0.09 | 0.01 | 0.01 |  |
|  | D/CD | 0.35 |  |  |  |  |  |  |  |  |  |
|  | D/D | 0.26 |  |  |  |  |  |  |  |  |  |
| 5 | D/CD | 0.60 | **0.54** | 0.01 | 0.02 | **0.34** | 0.01 | 0.07 | 0.01 | 0.01 |  |
|  | A/AC | 0.22 |  |  |  |  |  |  |  |  |  |
|  | A/D | 0.10 |  |  |  |  |  |  |  |  |  |
|  | A/C | 0.08 |  |  |  |  |  |  |  |  |  |
| 6 | A/A | 0.93 | **0.82** | 0.01 | 0.01 | 0.07 | 0.05 | 0.04 | 0.01 | 0.01 |  |
|  | A/AE | 0.07 |  |  |  |  |  |  |  |  |  |
| 7 | DCH/D | 0.47 | 0.01 | 0.08 | 0.09 | **0.82** | 0.003 | 0.003 | 0.003 | 0.003 |  |
|  | DCH/H | 0.17 |  |  |  |  |  |  |  |  |  |
|  | D/DH | 0.09 |  |  |  |  |  |  |  |  |  |
| 8 | DCH/C | 0.54 | 0.01 | 0.01 | **0.21** | **0.78** | 0.001 | 0.001 | 0.001 | 0.001 |  |
|  | DH/C | 0.19 |  |  |  |  |  |  |  |  |  |
|  | CH/C | 0.17 |  |  |  |  |  |  |  |  |  |
|  | H/C | 0.05 |  |  |  |  |  |  |  |  |  |
| 9 | A/A | 0.93 | **0.99** | 0.00 | 0.00 | 0.00 | 0.01 | 0.00 | 0.00 | 0.00 |  |
|  | AE/A | 0.03 |  |  |  |  |  |  |  |  |  |
| 10 | C/BH | 0.29 | 0.001 | 0.04 | 0.01 | **0.74** | 0.00 | 0.002 | 0.003 | 0.00 |  |
|  | C/H | 0.28 |  |  |  |  |  |  |  |  |  |
|  | CH/B | 0.15 |  |  |  |  |  |  |  |  |  |
| 11 | C/H | 0.22 | 0.01 | 0.14 | **0.67** | 0.13 | 0.01 | 0.02 | 0.01 | 0.01 |  |
|  | C/BH | 0.14 |  |  |  |  |  |  |  |  |  |
|  | H/H | 0.17 |  |  |  |  |  |  |  |  |  |
|  | H/B | 0.11 |  |  |  |  |  |  |  |  |  |
|  | CH/B | 0.11 |  |  |  |  |  |  |  |  |  |
| 12 | DH/H | 0.64 | 0.03 | 0.05 | 0.09 | **0.75** | 0.01 | 0.06 | 0.01 | 0.01 |  |
|  | DCH/C | 0.05 |  |  |  |  |  |  |  |  |  |
|  | DCH/H | 0.04 |  |  |  |  |  |  |  |  |  |
|  | DGH/H | 0.04 |  |  |  |  |  |  |  |  |  |
| 13 | D/G | 0.44 | 0.00 | 0.01 | 0.003 | **0.98** | 0.00 | 0.001 | 0.01 | 0.00 |  |
|  | D/D | 0.38 |  |  |  |  |  |  |  |  |  |
|  | D/DG | 0.05 |  |  |  |  |  |  |  |  |  |
