# Supplementary material for: Phylogeographic pattern of Rhizophora (Rhizophoraceae) reveals the importance of both vicariance and long-distance oceanic dispersal to modern mangrove distribution
Source: BMC Evol Biol. 2014 Apr 17;14:83. doi: 10.1186/1471-2148-14-83 (PMC4021169; doi:10.1186/1471-2148-14-83)
Supplement: Additional file 4 — GenBank accession number of Rhizophora samples included in this study. For each gene region, given sequence divergence among individuals of the same species from the same locality was less than 1%, one sequence was selected to represent a taxon from each locality. Asterisks denoted individuals used in Lo (2010). Alignment and tree were deposited in TreeBase (accession number 15468). [file 1471-2148-14-83-S4.docx]

**Additional file-5.** GenBank accession number of *Rhizophora* samples included in this study. For each gene region, given sequence divergence among individuals of the same species from the same locality was less than 1%, one sequence was selected to represent a taxon from each locality. Asterisks denoted individuals used in Lo (2010). Alignment and tree were deposited in TreeBase (accession number 15468).

| Taxon |  | Locality; Country | Label | Sample ID | GenBank accession number | | |
| --- | --- | --- | --- | --- | --- | --- | --- |
|  | |  |  |  | Ribosomal ITS | *trnH*-*rpl*2 | *trnS-trnG* |
| *Rhizophora apiculata* | |  |  |  |  |  |  |
|  |  | Cato River, Arnhem Bay; Australia | CAT | 004 | KJ194262 | KJ417099 | KJ417006 |
|  |  |  |  | 015* | HQ337910 | HQ337994 | HQ338050 |
|  |  |  |  | 016* | KJ194263 | KJ417100 | KJ417007 |
|  |  | Danitree River; Australia | DAI | 146 | HQ337909 | HQ337993 | HQ338049 |
|  |  |  |  | 150 | KJ194260 | KJ417097 | KJ417005 |
|  |  |  |  | 158* | KJ194261 | KJ417095 | KJ417003 |
|  |  | Embley River, Weipa; Australia | EMB | 031 | KJ194258 | KJ417102 | KJ417009 |
|  |  |  |  | 035* | HQ337911 | HQ337995 | HQ338051 |
|  |  |  |  | 045 | KJ194259 | KJ417101 | KJ417008 |
|  |  | Trinity Inlet, Carins; Australia | TRI | 030* | HQ338073 | HQ338005 | HQ338061 |
|  |  |  |  | 031* | KJ194257 | KJ417044 | KJ417040 |
|  |  |  |  | 033* | KJ194256 | KJ417098 | KJ417010 |
|  |  | Chuuk; Federated States of Micronesia | CHU | 009 | KJ194254 | KJ417116 | KJ417024 |
|  |  |  |  | 020* | HQ337918 | HQ338006 | HQ338062 |
|  |  |  |  | 024* | KJ194255 | KJ417117 | KJ417025 |
|  |  | Kosrae; Federated States of Micronesia | KOS | 014* | KJ194253 | KJ417121 | KJ417029 |
|  |  |  |  | 018* | HQ337919 | HQ338009 | HQ338065 |
|  |  | Yap; Federated States of Micronesia | YAP | 011 | KJ194251 | KJ417118 | KJ417026 |
|  |  |  |  | 012 | KJ194252 | KJ417119 | KJ417027 |
|  |  |  |  | 033* | HQ337920 | HQ338007 | HQ338063 |
|  |  | Guam | GUA | 020 | KJ194250 | KJ417120 | KJ417028 |
|  |  |  |  | 024* | HQ337924 | HQ338008 | HQ338064 |
|  |  | Iriomote Island; Japan | IRI | 027 | HQ337921 | HQ337996 | HQ338052 |
|  |  | North Sulawesi; Indonesia | IND | 040 | KJ194248 | KJ417106 | KJ417014 |
|  |  |  |  | 042 | KJ194249 | KJ417107 | KJ417015 |
|  |  |  |  | 043 | HQ337913 | HQ337999 | HQ338054 |
|  |  | Blue Lagoon, Cape Rachado; Malaysia | BLA | 019 | KJ194246 | KJ417103 | KJ417011 |
|  |  |  |  | 021 | KJ194247 | KJ417104 | KJ417012 |
|  |  |  |  | 022 | HQ337915 | HQ337997 | HQ338053 |
|  |  | Pulau Babi, TK Pelanduk; Malaysia | PBA | 018 | KJ194245 | KJ417108 | KJ417013 |
|  |  |  |  | 022 | HQ337923 | HQ337998 | HQ338055 |
|  |  | Pulau Burong; Malaysia | PBU | 019 | KJ194244 | KJ417105 | KJ417016 |
|  |  |  |  | 021 | HQ337914 | HQ338000 | HQ338056 |
|  |  | Sementa, Klang; Malaysia | SEM | 041 | KJ194243 | KJ417109 | KJ417017 |
|  |  |  |  | 043 | HQ337912 | HQ338001 | HQ338057 |
|  |  | Phang Nga Bay, Phunket; Thailand | PNB | 030 | HQ337922 | HQ338002 | HQ338058 |
|  |  |  |  | 183 | KJ194241 | KJ417110 | KJ417018 |
|  |  |  |  | 193 | KJ194242 | KJ417111 | KJ417019 |
|  |  | Panay Island; Philippines | PHI | 043 | KJ194239 | KJ417112 | KJ417020 |
|  |  |  |  | 044 | HQ337916 | HQ338003 | HQ338059 |
|  |  |  |  | 047 | KJ194240 | KJ417113 | KJ417021 |
|  |  | West coast; Sri Lanka | SRI | 022* | HQ337917 | HQ338004 | HQ338060 |
|  | |  |  | 023* | KJ194237 | KJ417114 | KJ417022 |
|  | |  |  | 024* | KJ194238 | KJ417115 | KJ417023 |
| *Rhizophora mangle* | |  |  |  |  |  |  |
|  |  | Kahalu, eastern coast of Oahu; Hawaii, USA | KAH | 075 | KJ194269 | KJ417122 | KJ417030 |
|  |  |  |  | 095* | HQ337957 | HQ338011 | HQ338067 |
|  |  |  |  | 105 | KJ194270 | KJ417123 | KJ417031 |
|  |  | Waipahu, southern coast of Oahu; Hawaii, USA | WAI | 109 | KJ194268 | KJ417125 | KJ417033 |
|  |  |  |  | 110 | HQ337956 | HQ338010 | HQ338066 |
|  |  |  |  | 131 | KJ194267 | KJ417124 | KJ417032 |
|  |  | Atlantic coast, Panama | APA | 018* | HQ337954 | HQ338012 | HQ338068 |
|  |  |  |  | 022 | KJ194264 | KJ417126 | KJ417034 |
|  |  | Pacific coast, Panama | PPA | 001 | KJ194265 | KJ417045 | KJ417041 |
|  |  |  |  | 002* | HQ337955 | HQ338014 | HQ338070 |
|  |  | Pacific coast, Mexico | PMX | 011 | HQ337958 | HQ338013 | HQ338069 |
|  | |  |  | 014 | KJ194266 | KJ417127 | KJ417035 |
| *Rhizophora mucronata* | |  |  |  |  |  |  |
|  |  | Danitree River; Australia | DAI | 083 | KJ194236 | KJ417076 | KJ416983 |
|  |  |  |  | 184* | KJ194235 | KJ417077 | KJ416984 |
|  |  |  |  | 192* | HQ337953 | HQ337964 | HQ338022 |
|  |  | Trinity Inlet, Carins; Australia | TRI | 028* | HQ337942 | HQ337965 | HQ338023 |
|  |  |  |  | 030 | KJ194233 | KJ417078 | KJ416985 |
|  |  |  |  | 031 | KJ194234 | KJ417043 | KJ417039 |
|  |  | Gazi Bay; Kenya | GAZ | 002 | HQ337949 | HQ337974 | HQ338030 |
|  |  |  |  | 004 | KJ194232 | KJ417091 | KJ416999 |
|  |  |  |  | 005 | KJ194231 | KJ417092 | KJ417000 |
|  |  | Mida Creek; Kenya | MID | 002 | HQ337948 | HQ337975 | HQ338031 |
|  |  |  |  | 011 | KJ194229 | KJ417094 | KJ417001 |
|  |  |  |  | 013 | KJ194230 | KJ417093 | KJ417002 |
|  |  | Kosrae; Federated States of Micronesia | KOS | 043* | HQ337945 | HQ337968 | HQ338024 |
|  |  |  |  | 046 | KJ194228 | KJ417081 | KJ416989 |
|  |  | Yap; Federated States of Micronesia | YAP | 064 | KJ194226 | KJ417079 | KJ416987 |
|  |  |  |  | 066* | HQ337944 | HQ337967 | HQ338021 |
|  |  |  |  | 070 | KJ194227 | KJ417080 | KJ416988 |
|  |  | Iriomote Island; Japan | IRI | 028 | HQ337943 | HQ337966 | HQ338020 |
|  |  |  |  | 030 | KJ194225 | KJ417131 | KJ416986 |
|  |  | North Sulawesi; Indonesia | IND | 002 | KJ194224 | KJ417086 | KJ416994 |
|  |  |  |  | 004 | HQ337952 | HQ337971 | HQ338027 |
|  |  |  |  | 019 | KJ194223 | KJ417087 | KJ416995 |
|  |  | Sementa Klang; Malaysia | SEM | 008 | HQ337951 | HQ337972 | HQ338028 |
|  |  |  |  | 011 | KJ194222 | KJ417088 | KJ416996 |
|  |  | Phang Nga Bay, Phunket; Thailand | PNB | 051 | HQ337950 | HQ337973 | HQ338029 |
|  |  |  |  | 060 | KJ194221 | KJ417089 | KJ416997 |
|  |  |  |  | 062 | KJ194220 | KJ417090 | KJ416998 |
|  |  | Panay Island; Philippines | PHI | 002 | HQ337947 | HQ337970 | HQ338026 |
|  |  |  |  | 005 | KJ194219 | KJ417085 | KJ416993 |
|  |  |  |  | 022 | KJ194218 | KJ417084 | KJ416992 |
|  |  | West coast; Sri Lanka | SRI | 002* | HQ337946 | HQ337969 | HQ338025 |
|  | |  |  | 003* | KJ194216 | KJ417082 | KJ416990 |
|  | |  |  | 006* | KJ194217 | KJ417083 | KJ416991 |
| *Rhizophora racemosa* | |  |  |  |  |  |  |
|  |  | Pacific coast, Panama | PPA | 001 | KJ194273 | KJ417046 | KJ417042 |
|  | |  |  | 002 | KJ194274 | KJ417128 | KJ417036 |
|  | |  |  | 004 | HQ337960 | HQ338016 | HQ338072 |
| *Rhizophora samoensis* | |  |  |  |  |  |  |
|  |  | VitiLevu Island; Fiji | VIT | 018* | KJ194272 | KJ417130 | KJ417038 |
|  | |  |  | 019* | KJ194271 | KJ417129 | KJ417037 |
|  | |  |  | 020* | HQ337959 | HQ338015 | HQ338071 |
| *Rhizophora stylosa* | |  |  |  |  |  |  |
|  |  | Cato River, Arnhem Bay; Australia | CAT | 001 | KJ194191 | KJ417055 | KJ416962 |
|  |  |  |  | 002* | KJ194192 | KJ417054 | KJ416961 |
|  |  |  |  | 003* | HQ337926 | HQ337979 | HQ338035 |
|  |  | Danitree River; Australia | DAI | 189 | KJ194188 | KJ417048 | KJ416955 |
|  |  |  |  | 205 | KJ194187 | KJ417047 | KJ416954 |
|  |  |  |  | 208* | HQ337940 | HQ337976 | HQ338032 |
|  |  |  |  | 239* | KJ194186 | KJ417049 | KJ416956 |
|  |  | Embley River, Weipa; Australia | EMB | 001* | HQ337925 | HQ337978 | HQ338034 |
|  |  |  |  | 002* | KJ194193 | KJ417052 | KJ416959 |
|  |  |  |  | 003 | KJ194194 | KJ417053 | KJ416960 |
|  |  | Trinity Inlet, Carins; Australia | TRI | 001 | KJ194189 | KJ417050 | KJ416957 |
|  |  |  |  | 003* | HQ337929 | HQ337977 | HQ338033 |
|  |  |  |  | 004 | KJ194190 | KJ417051 | KJ416958 |
|  |  | Shoalwater Bay, Queensland; Australia | SWB | 001 | HQ337927 | HQ337992 | HQ338048 |
|  |  |  |  | 002 | KJ194209 | KJ417056 | KJ416963 |
|  |  | Chuuk; Federated States of Micronesia | CHU | 003* | HQ337931 | HQ337980 | HQ338037 |
|  |  |  |  | 004* | KJ194206 | KJ417059 | KJ416966 |
|  |  |  |  | 005 | KJ194205 | KJ417060 | KJ416967 |
|  |  | Kosrae; Federated States of Micronesia | KOS | 001* | KJ194204 | KJ417058 | KJ416964 |
|  |  |  |  | 008* | HQ337930 | HQ337981 | HQ338036 |
|  |  |  |  | 013 | KJ194203 | KJ417057 | KJ416965 |
|  |  | Yap; Federated States of Micronesia | YAP | 004* | HQ337933 | HQ337991 | HQ338047 |
|  |  |  |  | 020 | KJ194208 | KJ417061 | KJ416970 |
|  |  |  |  | 022 | KJ194207 | KJ417062 | KJ416971 |
|  |  | Guam | GUA | 001* | HQ337932 | HQ337982 | HQ338038 |
|  |  |  |  | 005* | KJ194211 | KJ417063 | KJ416968 |
|  |  |  |  | 006 | KJ194210 | KJ417064 | KJ416969 |
|  |  | Iriomote Island; Japan | IRI | 001 | KJ194212 | KJ417065 | KJ416972 |
|  |  |  |  | 025 | HQ337937 | HQ337983 | HQ338039 |
|  |  | North Sulawesi; Indonesia | IND | 021 | KJ194200 | KJ417067 | KJ416974 |
|  |  |  |  | 025 | HQ337941 | HQ337986 | HQ338042 |
|  |  | Blue Lagoon, Cape Rachado; Malaysia | BLA | 003 | HQ337935 | HQ337985 | HQ338041 |
|  |  |  |  | 004 | KJ194199 | KJ417066 | KJ416973 |
|  |  | Pulau Babi, TK Pelanduk; Malaysia | PBA | 009 | HQ337936 | HQ337987 | HQ338045 |
|  |  |  |  | 020 | KJ194198 | KJ417072 | KJ416979 |
|  |  |  |  | 022 | KJ194197 | KJ417073 | KJ416980 |
|  |  | Pulau Burong; Malaysia | PBU | 001 | HQ337939 | HQ337989 | HQ338043 |
|  |  |  |  | 002 | KJ194195 | KJ417068 | KJ416975 |
|  |  |  |  | 003 | KJ194196 | KJ417069 | KJ416976 |
|  |  | Panay Island; Philippines | PHI | 026 | HQ337938 | HQ337988 | HQ338044 |
|  |  |  |  | 027 | KJ194201 | KJ417071 | KJ416978 |
|  |  |  |  | 028 | KJ194202 | KJ417070 | KJ416977 |
|  |  | Taiwan | TAW | 068 | HQ337934 | HQ337984 | HQ338040 |
|  |  |  |  | 070 | KJ194213 |  |  |
|  |  | Vitilevu Island; Fiji | VIT | 001* | HQ337928 | HQ337990 | HQ338046 |
|  | | |  | 002* | KJ194215 | KJ417074 | KJ416981 |
|  | | |  | 003* | KJ194214 | KJ417075 | KJ416982 |
| *Bruguiera gymnorrhiza* (outgroup) | | |  |  |  |  |  |
|  |  | Vitilevu Island; Fiji | VIT |  | HQ337961 | HQ338017 | - |
|  |  | Phang Nga Bay, Phunket; Thailand | PNB |  | HQ337963 | HQ338019 | - |
|  |  | Pulau Burong; Malaysia | PBU |  | HQ337962 | HQ338018 | - |
