# Supplementary material for: Phylogeographic pattern of Rhizophora (Rhizophoraceae) reveals the importance of both vicariance and long-distance oceanic dispersal to modern mangrove distribution
Source: BMC Evol Biol. 2014 Apr 17;14:83. doi: 10.1186/1471-2148-14-83 (PMC4021169; doi:10.1186/1471-2148-14-83)
Supplement: Additional file 5 — Sequences of the 16 primers used for PCR amplification of inter-simple sequence repeat (ISSR). [file 1471-2148-14-83-S5.doc]

**Additional file** **2**. Sequences of the 16 primers used for PCR amplification of inter-simple sequence repeat (ISSR)

| ISSR | UBC Primer No. | Nucleotide Sequence^a^ |
| --- | --- | --- |
|  | 807 | AGA GAG AGA GAG AGA GT |
|  | 810 | GAG AGA GAG AGA GAG AG |
|  | 811 | GAG AGA GAG AGA GAG AC |
|  | 817 | CAC ACA CAC ACA CAC AA |
|  | 818 | CAC ACA CAC ACA CAC AG |
|  | 823 | TCT CTC TCT CTC TCT CG |
|  | 825 | ACA CAC ACA CAC ACA CT |
|  | 834 | AGA GAG AGA GAG AGA GYT |
|  | 835 | AGA GAG AGA GAG AGA GYC |
|  | 836 | AGA GAG AGA GAG AGA GCT C |
|  | 844 | CTC TCT CTC TCT CTC TAG C |
|  | 846 | CAC ACA CAC ACA CAC AAG T |
|  | 847 | CAC ACA CAC ACA CAC ARC |
|  | 889 | DBDACACAC ACA CAC AC |
|  | 890 | GTG TGT GTG TGT GTA CG |
|  | 891 | TGT GTG TGT GTG TGA CT |

^a^ Y: C or T; R: A or G; D: A or G or T; B: C or G or T.
